# Exploring the Association between Personality Traits, Symptom Burden, and Return to Work after Mild-to-Moderate Traumatic Brain Injury

**DOI:** 10.3390/jcm12144654

**Published:** 2023-07-13

**Authors:** Benedikte Å. Madsen, Silje C. R. Fure, Nada Andelic, Daniel Løke, Marianne Løvstad, Cecilie Røe, Emilie Isager Howe

**Affiliations:** 1 Department of Physical Medicine and Rehabilitation, Oslo University Hospital, 0424 Oslo, Norway; siljfu@ous-hf.no (S.C.R.F.); nadand@ous-hf.no (N.A.); cecilie.roe@medisin.uio.no (C.R.); emihow@ous-hf.no (E.I.H.); 2 Institute of Clinical Medicine, Faculty of Medicine, University of Oslo, 0318 Oslo, Norway; 3 Research Center for Habilitation and Rehabilitation Models and Services (CHARM), Institute of Health and Society, University of Oslo, 0373 Oslo, Norway; 4 Department of Research, Sunnaas Rehabilitation Hospital Trust, 1453 Nesoddtangen, Norway; dloeke@gmail.com (D.L.); marianne.lovstad@sunnaas.no (M.L.); 5 Department of Psychology, Faculty of Social Sciences, University of Oslo, 0373 Oslo, Norway

**Keywords:** brain injury, concussion, neuroticism, openness to experience, conscientiousness, personality inventory, mental health, post-concussion symptoms, return to work

## Abstract

Approximately 30% of individuals with mild traumatic brain injury (mTBI) experience persistent post-concussion symptoms (PPCS). Personality factors have been linked to PPCS, yet, the association between personality traits and outcomes after mTBI is poorly understood. The aim of this study was to evaluate the association between personality traits, PPCS, and return to work (RTW) in patients with mild-to-moderate traumatic brain injury (TBI). Data from eighty-seven participants with mild-to-moderate TBI were analyzed. Sociodemographic, injury, and work characteristics and depressive symptoms were recorded 2–3 months post-injury. Personality traits were measured using the NEO Five-Factor Inventory-3. PPCS and RTW were assessed 15 months post-injury. Multiple linear regression models were performed. The factors associated with more severe PPCS were female sex, higher levels of neuroticism, openness to experience and conscientiousness, extra-cranial injuries, and depressive symptoms. The factors associated with lower RTW were female sex, higher levels of neuroticism, and conscientiousness. However, after controlling for PPCS, personality traits were no longer significantly associated with RTW. In conclusion, specific personality traits were associated with more severe PPCS and may be indirectly associated with RTW via PPCS. Hence, personality traits may be important to assess to identify patients at risk of less favorable outcomes after mild-to-moderate TBI.

## 1. Introduction

The majority (70–90%) of traumatic brain injuries (TBIs) are classified as mild, whereas around 8% are moderate [1,2]. Approximately one-third of patients who sustain a mild or moderate traumatic brain injury report somatic, emotional, and cognitive symptoms that persist beyond 3 months post-injury [3,4]. These symptoms are commonly referred to as persistent post-concussion symptoms (PPCS) [5]. Injury-related variables alone have limited predictive value for symptom burden after mild traumatic brain injury (mTBI) [6,7]. Research has shown that prolonged symptom recovery is associated with pre-injury mental health problems [8,9], injury-related emotional distress [8,10,11], and concurrent emotional distress [12,13]. It is important to gain more knowledge about non-injury factors that predict mTBI recovery, such as personality traits that may influence the development or maintenance of symptoms [14].

Although the majority of individuals who sustain mTBI return to work (RTW) within 3–6 months post-injury [15], up to 20% of those with mTBI and 50% with moderate TBI continue to experience difficulties that affect work participation 6–12 months after injury [16,17]. Owing to the high frequency of mTBI, this constitutes a substantial number of individuals and highlights the profound economic and psychosocial consequences [18]. An individual’s inability to RTW after TBI is multifactorial, and potential barriers include injury characteristics, lack of employment assistance [18], and pre- and post-injury psychological problems [19]. De Koning et al. [20] found that posttraumatic complaints and signs of psychological distress early after injury were predictors of both short- and long-term RTW. Early identification of individuals at risk of failure to RTW is important for understanding barriers to work and providing targeted interventions [20].

Personality appears to influence recovery after mTBI [21]. Personality traits can be defined as relatively stable, consistent, and enduring internal characteristics that are inferred from a pattern of behaviors, attitudes, feelings, and habits in an individual [22]. The most influential theory for human personality is the five-factor model (FFM), which identifies five broad personality domains (i.e., neuroticism, extraversion, openness to experience, agreeableness, and conscientiousness) [23,24]. The FFM is widely identified across cultures [25,26] and has strong predictive value for other health and life outcomes [27,28]. The literature on personality and mTBI has mainly focused on the association between neuroticism (i.e., the stable tendency toward negative affect [29]), symptom burden, and psychological distress. Higher levels of neuroticism have been linked to increased post-concussion symptom reporting after mTBI [30,31,32,33], as well as more symptoms of anxiety and depression and worse functional outcomes following mTBI [34,35]. Moreover, personality traits associated with negative affectivity have been shown to indirectly predict PPCS through the enhancement of acute somatic complaints [36]. To our knowledge, only two studies have assessed the association between post-concussion symptoms and personality traits beyond neuroticism [32,33]. Skandsen et al. [32] found an association between higher levels of openness to experience and PPCS. Contrarily, Summerell and colleagues [33] found that lower levels of openness to experience and conscientiousness were associated with more PPCS. To better identify protective factors, patients at risk, and response to treatment in clinical practice, there is a need for more research on personality traits and how they may influence other outcomes (e.g., RTW) after mTBI.

The association between personality traits and symptom burden (i.e., PPCS and depressive symptoms) after mTBI highlights how bio-medical and psychological processes may interact to influence recovery. Still, the role of personality traits in RTW- an important outcome of recovery after mTBI, needs further assessment. The aim of this study was thus to explore the association between personality traits, PPCS, and RTW in patients with mild-to-moderate TBI at 15 months post-injury. There were two main reasons for choosing the 15-month follow-up time point. Firstly, as up to 20% of individuals who sustain a mTBI experience problems that affect work participation 12 months post-injury [16], it is relevant to explore factors associated with failure to RTW in the longer term. Secondly, the Norwegian social security system provides full sick leave compensation for the first year of sick-listing, which might affect the motivation to RTW, and it is therefore of interest to investigate RTW beyond this time point [37]. Based on the existing literature [31,33,35], we hypothesized that higher levels of neuroticism and depressive symptoms, and lower levels of openness to experience and conscientiousness, would be associated with more severe PPCS and lower RTW. Additionally, we expected that more severe PPCS would be associated with lower RTW.

## 2. Materials and Methods

### 2.1. Study Design

This study was based on a previous randomized controlled trial (RCT) [38], which compared the effectiveness of a combined cognitive and vocational intervention to multidisciplinary treatment as usual on RTW and other outcomes after mild-to-moderate TBI. Participants received either the study intervention or treatment as usual for a total of 6 months, and the main results have been published previously [39,40]. As there were no statistically significant between-group differences in RTW or PPCS at 15 months post-injury, the participants were analyzed as one cohort in this study. The RCT was approved by the Regional Committee for Medical and Health Research Ethics in South-Eastern Norway (#2016/2038). Notification of amendment for secondary analyses was approved on 28 February 2022 (6481).

### 2.2. Participants and Inclusion Criteria

One hundred and sixteen individuals were included in the original RCT. They were referred between July 2017 and April 2019 from the neurosurgical department, the emergency room, or by general practitioners to a specialized TBI outpatient clinic at Oslo University Hospital (OUH), Norway. In this study, 87 of the 116 individuals were included since 29 individuals had missing responses on the NEO Five-Factor Inventory-3 (NEO-FFI-3) [41,42], which was used to evaluate personality traits. The eligibility criteria for the original RCT were as follows: mild-to-moderate TBI 2–3 months previously as assessed by a Glasgow Coma Scale (GCS) score of 10–15, loss of consciousness (LOC) for <24 h, and posttraumatic amnesia (PTA) for <7 days [43], age of 18–60 years, residency in Oslo or Akershus County and employment at a minimum of 50% position at the time of injury. Further, at inclusion, the participants were sick-listed 50% or more relative to their pre-injury employment percentage due to PPCS as assessed using the Rivermead Post-Concussion Symptoms Questionnaire (RPQ) [44]. The criteria developed by the American Congress of Rehabilitation Medicine [45] were used to establish mTBI by reviewing medical records or established at the time of screening for study eligibility. The exclusion criteria were severe psychiatric or neurological illness, active substance abuse, and insufficient Norwegian language skills.

### 2.3. Procedures

After written informed consent for participation was obtained, sociodemographic, injury- and work-related information was collected during an interview. Further, the participants underwent a baseline assessment (2–3 months after injury) of self-reported post-concussion and emotional symptoms. Clinical characteristics were collected from medical records and self-reports. Either a clinical psychologist or a medical doctor performed the baseline assessment at the outpatient clinic at the Department of Physical Medicine and Rehabilitation, OUH. Follow-up assessments were conducted at the outpatient clinic 3, 6, and 12 months after study inclusion (i.e., approximately 6, 9, and 15 months post-injury).

### 2.4. Variables

#### 2.4.1. Sociodemographic and Work-Related Factors

The following variables were included: age (years), sex (male/female), educational level (years), relationship status (married/cohabitating vs. single/other), employment status (full- or part-time position), and occupation type. We defined occupation type as white (non-manual labor) or blue collar (manual labor) and further recorded if the participants worked in the private or public sector and whether they had permanent or temporary positions.

#### 2.4.2. Personality Traits 

To measure personality traits in this study, we used the NEO-FFI-3 [41,42], assessed at 15 months post-injury. The NEO-FFI-3 is a 60-item version of the NEO Personality Inventory-3 (NEO-PI-3) that measures five dimensions of personality. Individuals can score high or low in each domain: neuroticism (e.g., the tendency to experience negative affect versus emotional stability); extraversion (e.g., sociability, assertiveness, excitement-seeking, and optimism versus reserved, independent, and even-paced); openness to experience (e.g., active imagination, attentiveness to inner feelings, and intellectual curiosity versus conventional and conservative); agreeableness (e.g., trustful, altruistic, and modest versus skeptical and antagonistic); and conscientiousness (e.g., purposeful, determined, and organized versus unreliable and spontaneous). There are 12 statements assessing each dimension, which are rated on a 5-point Likert scale ranging from 0 (strongly disagree) to 4 (strongly agree). Raw scores are converted to standardized scores (T-scores) using sex-corrected norms collected from a Norwegian population-based sample [42] with a mean of 50 (standard deviation [SD] = 10). Higher scores indicate higher levels of the personality trait measured. T-scores of 56 or higher are considered high; T-scores ranging from 45 to 55 are considered average; and T-scores of 44 or lower are considered low. The NEO-FFI-3 is shorter than the NEO-PI-3 and does not measure the six underlying facets of each domain, but it is more manageable and time efficient. The questionnaire has been translated into Norwegian [42] and has been validated in the Scandinavian context [24,46].

#### 2.4.3. Injury-Related Factors

The presence of traumatic intracranial findings on CT/MRI was dichotomized into yes/no. TBI severity was classified as mild or moderate using the GCS [47], LOC, and PTA (i.e., mTBI: GCS 13–15, LOC 0–30 min and PTA < 1 day; moderate TBI: GCS 10–12, LOC < 24 h and PTA < 7 days) [43]. Extra-cranial injuries were recorded according to the affected body part and severity using the Abbreviated Injury Scale [48] (dichotomized into yes/no).

#### 2.4.4. Emotional Symptoms

The Patient Health Questionnaire-9 (PHQ-9) [49] was used to measure depressive symptoms at baseline (2–3 months post-injury). It consists of nine items, which are scored from 0 (‘not at all’) to 3 (‘nearly every day’). A score of 10 or higher indicates moderate-to-severe depressive symptoms.

#### 2.4.5. Persistent Post-Concussion Symptoms

PPCS was measured at baseline and 15 months post-injury using the RPQ [44]. PPCS measured at 15 months post-injury was used as the first main outcome variable. The RPQ covers somatic, cognitive, and emotional symptoms. Sixteen post-concussion symptoms are rated on a 5-point Likert scale ranging from 0 to 4, wherein 0 indicates ‘not experienced’; 1 ‘no more of a problem’; 2 ‘mild problem’; 3 ‘moderate problem’; and 4 ‘severe problem’. All scores of 1 (indicating that the problem was the same as before the injury) were removed.

#### 2.4.6. Return to Work

RTW was the second main outcome variable in this study, defined as the work percentage (0–100%) at 15 months post-injury. Information on RTW was based on patients’ self-reports and calculated relative to their pre-injury work levels.

### 2.5. Statistical Analyses

Stata version 16 was used to analyze the data [50]. Descriptive statistics for baseline characteristics are presented as means with SDs or proportions with percentages. We performed additional sensitivity analyses comparing the baseline characteristics of patients who completed the NEO-FFI-3 (N = 87) to those who did not (N = 29) (see Table A1 in Appendix A), revealing no statistically significant differences with the exception of years of education. Two separate multivariable linear regression analyses were performed: one with PPCS as an outcome and one with RTW as an outcome, both measured at 15 months post-injury. The independent variables were chosen on the basis of expert knowledge and previous findings. All independent variables (age, sex, educational level, relationship status, all five personality traits, CT/MRI findings, injury severity, extra-cranial injuries, employment status, occupation type, working in the private or public sector, temporary or permanent position, PPCS and PHQ-9 at baseline) were initially included in the models and then reduced using manual backward elimination to optimize model fit. Moreover, to control for possible effects of the treatment provided in the original RCT that this study is based on, a variable representing intervention vs. control group was included in each regression model and revealed no significant association with the outcomes. The second analysis (with RTW as the outcome variable) included two steps. Step one, with the above-mentioned independent variables, and step two, where PPCS at 15 months post-injury was included as an independent variable to assess the association between personality traits, PPCS, and RTW. We also tested for interactions between personality traits, but none of the interactions appeared to be associated with the outcome variables and were therefore not included in our final analyses. Because only 87 participants were included, there was a limited number of independent variables that could be added, but at least one variable per category (sociodemographic and injury- and work-related characteristics and symptom- burden) was kept in the final models. For each step, an assessment of the Akaike information criterion was performed. The amount of variance in PPCS and RTW explained by the model was represented by R^2^ and adjusted R^2^. Multicollinearity was checked using a variable inflation factor. The normality of the residuals was controlled using a Q-Q plot. The models were run with 1,000 bootstrap repetitions, and statistical significance was set at *p* < 0.05.

## 3. Results

### 3.1. Baseline Characteristics of the Participants

Table 1 shows participant characteristics at baseline. The participants’ mean age was 43 years, and 60% were women. The predominant cause of injury was falls, followed by exposure to inanimate objects and traffic accidents. Most participants (94%) were classified as having mTBI, and 24% had evidence of traumatic intra-cranial findings on CT/MRI. Additionally, 84% of the participants were sick-listed between 80 and 100%. The mean RPQ and PHQ-9 scores at baseline were twenty-eight and nine, respectively; 44% had a score of ten or higher on PHQ-9, indicating clinically significant depressive symptoms. The scores for each of the five personality traits in the sample were normally distributed (Table 1). 

### 3.2. Association between Personality Traits and Persistent Post-Concussion Symptoms

In the best-fitting model, the variables significantly associated with more severe PPCS at 15 months post-injury were female sex, higher levels of neuroticism, openness to experience and conscientiousness, extra-cranial injuries, and depressive symptoms at baseline (Table 2). The regression coefficients indicated that the strength of the association between conscientiousness and PPCS was approximately twice the magnitude (0.62) compared to neuroticism (0.36) or openness to experience (0.31). The model explained 52% of the variance in PPCS at 15 months post-injury. Bootstrapping analysis supported all significant associations in the model (female sex: 8.2, 95% CI = 4 to 12, *p* = 0.00; neuroticism: 0.4, 95% CI = 0.1 to 0.6, *p* = 0.01; openness: 0.3, 95% CI = 0.1 to 0.5, conscientiousness: 0.6, 95% CI = 0.3 to 0.9, *p* = 0.00; extra-cranial injuries: 4.6, 95% CI = 0.5 to 8.7, *p* = 0.03; depressive symptoms: 1.1, 95% CI = 0.7 to 1.6, *p* = 0.00). Extraversion and agreeableness were left out of the final models because they were not significantly associated with PPCS, and excluding them yielded a better-fitting model. Injury severity did not contribute significantly, but the quality of the model was degraded when excluded. PPCS at baseline predicted more severe PPCS at 15 months post-injury but was left out of the final model to limit the number of variables and because including depressive symptoms yielded a better-fitting model.

### 3.3. Association between Personality Traits and Return to Work

In the best-fitting model, the variables significantly associated with lower RTW at 15 months post-injury were female sex and higher levels of neuroticism and conscientiousness. Being employed in the public sector was significantly associated with higher RTW (Table 3, step I). The model explained 19% of the variance in RTW. The bootstrapping analysis supported all significant associations in this model (female sex: −22, 95% CI = −37 to −7, *p* = 0.00; neuroticism: −1, 95% CI = −2 to −0.3, *p* = 0.01; conscientiousness: −1, 95% CI = −2 to −0.02, *p* = 0.05; employed in the public sector: 15, 95% CI = 0.8 to 29, *p* = 0.04). Injury severity, extra-cranial injuries, and depressive symptoms did not contribute significantly but were kept because of better model fit.

Adding PPCS measured at 15 months post-injury to the model (Table 3, step II) yielded a significant association between more severe PPCS and lower RTW (PPCS: −1.3, 95% CI = −2 to 0.4, *p* = 0.01). In addition, working in the public sector was significantly associated with higher RTW in step II (employed in the public sector: 14, 95% CI = 1 to 28, *p* = 0.04). The explained variance increased from 19% in step I to 27% in step II (see Table 3). Additionally, when PPCS was added in step II, the association between neuroticism and conscientiousness and RTW was no longer significant.

## 4. Discussion

The aim of this study was to explore the association between personality traits, PPCS, and RTW after mild-to-moderate TBI. This study found that higher levels of neuroticism, openness to experience, and conscientiousness, in addition to female sex, extra-cranial injuries, and depressive symptoms, were significantly associated with more severe PPCS at 15 months post-injury. Moreover, higher levels of neuroticism and conscientiousness, in addition to female sex, were significantly associated with lower RTW at 15 months post-injury. In addition, working in the public sector was significantly associated with higher RTW at 15 months post-injury. More severe PPCS was also significantly associated with lower RTW at 15 months post-injury, and when added to the final model, PPCS outperformed the earlier-mentioned factors and increased the explained variance.

To date, there is limited research on personality traits other than neuroticism and its association with PPCS [33,36,51]. In line with previous research and our hypothesis, we found that higher levels of neuroticism were associated with more severe PPCS at 15 months post-injury. High levels of neuroticism are characterized by being prone to experiencing negative emotions, such as anxiety, irritability, sadness, anger, and self-consciousness [29]. Individuals with high levels of neuroticism are more likely to report post-concussion symptoms following mTBI [31,33,36] and are more susceptible to developing symptoms of depression and anxiety [33,35,52]. This information can support the hypothesis that internalizing personality traits, such as neuroticism, can influence mTBI recovery through the enhancement of acute somatic complaints [36].

Interestingly, we also found that higher levels of conscientiousness were associated with more severe PPCS at 15 months post-injury. This was in contrast to our hypothesis and the findings by Summerell and colleagues [33], who found that higher levels of conscientiousness predicted lower PPCS 6–12 weeks after mTBI. Individuals who score high on conscientiousness are considered motivated, self-disciplined, efficient, hard-working, and organized [53], characteristics that are generally considered positive attributes. However, it has been suggested that these characteristics are not inherently positive or negative but rather dependent on the situation or context [54]. In the longitudinal study by Boyce and colleagues [55], the moderating effect of conscientiousness on well-being after unemployment was assessed in 9570 previously employed individuals. The authors found that individuals who scored high on conscientiousness experienced a significant decrease in life satisfaction following unemployment when compared with individuals who scored low. Kok et al. [56] explored the impact of conscientiousness on absenteeism in employees with affective disorders and found that individuals who were highly conscientious and had an anxiety or comorbid disorder had significantly higher odds of long-term absenteeism than average conscientious employees with comorbidity or anxiety. The authors hypothesized that highly conscientious individuals may be more vulnerable to these conditions owing to a stronger negative reaction to adverse life events and a higher sense of responsibility. Although not directly comparable, a possible explanation for our findings can be that this hard-working and conscientious group experiences difficulty coping with symptoms (e.g., reduced concentration or mental fatigue) and the consequences of the injury. Hence, they experience a sense of loss of control of their lives [57], which can lead to emotional distress and depressive symptoms.

In accordance with our findings, openness to experience has previously been shown to be associated with more severe post-concussion symptoms [32]. This is, however, in contrast to our hypothesis and the study by Summerell et al. [33], which found that higher levels of openness to experience predicted lower PPCS 6–12 weeks after mTBI. Individuals with high scores on openness to experience tend to be creative, aware of their own feelings, enjoy new experiences, and challenge authorities [58]. They are sensitive to mental and physical experiences, which may contribute to a higher symptom burden after mTBI. Higher scores on openness to experience have also been linked to higher education [59] and, likely, more cognitively demanding professions which might also contribute to higher symptom burden after mTBI. Nevertheless, there are still very few studies regarding openness to experience and recovery after mTBI, and the underlying mechanisms need further investigation.

This study showed that having sustained extra-cranial injuries and experiencing depressive symptoms at baseline were associated with more severe PPCS at 15 months post-injury. This finding supports previous reports that depressive symptoms and emotional distress are important factors in prolonged symptom recovery after TBI [31] and that injury-related variables alone have limited predictive value in symptom burden after mTBI [6,7]. Moreover, we found that female sex was significantly associated with more severe PPCS at 15 months post-injury. This result is in line with data from a recent study exploring sex and gender differences after TBI in a large European patient cohort [60]: Women had poorer outcomes and more severe self-reported post-concussion symptoms than men 6 months post-mTBI. Although biological differences, aspects of gender roles, and identity have been proposed as potential explanations for the observed sex and gender differences in the outcomes after TBI, more research is needed to shed light on this topic. However, notably, women were overrepresented in this study sample. The gender distribution, however, reflects patients who receive follow-up at the specialized outpatient clinic from which the participants were recruited [39], with one possible explanation being gender differences in help-seeking behavior and healthcare utilization. We also found that working in the public sector was significantly associated with higher RTW at 15 months post-injury. Our research group has previously reported similar results [61], and we hypothesize that this might be due to a greater sense of job security and access to work accommodations.

Our hypothesis regarding the association between personality traits and RTW was partly confirmed: Neuroticism and conscientiousness were significantly associated with lower RTW at 15 months post-injury. We also found that more severe PPCS was associated with lower RTW. To our knowledge, no previous studies have investigated associations between personality traits, PPCS, and RTW in a mild-to-moderate TBI population. Nevertheless, studies from other patient populations have shown associations between some personality traits and RTW. For example, a systematic review and meta-analysis by Fisker et al. [62] demonstrated that neuroticism and openness to experience predicted a lower probability of RTW among people on sick leave due to common mental disorders, which is in line with our findings. Regarding symptom burden and RTW, Yue et al. [63] found that post-concussion and emotional symptoms predicted the inability of previously employed individuals to RTW at 6 months after mTBI. De Koning et al. [20] found that posttraumatic complaints, extra-cranial injuries, and psychological distress were predictive of a lower work percentage at 12 months after injury. Interestingly, when we added PPCS to the final model (Table 3, step II), neuroticism and conscientiousness were no longer significantly associated with RTW. This might suggest that specific personality traits are indirectly associated with RTW via their effects on PPCS. Indirect effects, such as mediation and moderation, should be investigated further to obtain clearer insights into the complexity of associations between personality traits, PPCS, and RTW in patients with mTBI.

This study has limitations that should be acknowledged. Personality traits were measured retrospectively (i.e., at 15 months post-injury). Moreover, we did not include personality ratings from next of kin and, therefore, cannot definitely rule out whether post-injury functioning and experiences influenced self-reported personality. However, in contrast to studies assessing personality after severe TBI [64], personality characteristics have been found to not change and to remain stable up to 2 years after mTBI [35,65]. We, therefore, argue that the personality measure is valid despite being measured retrospectively. Next, the study participants were recruited in accordance with specific criteria, such as experiencing persisting symptoms interfering with work participation at 2–3 months post-injury. Thus, the findings may not generalize to all individuals with mTBI. Moreover, we also included patients with moderate TBI, but they represented a small proportion of the total study sample, and the results should therefore be interpreted with caution with regard to patients with moderate TBI. However, the sample is representative of the sub-group of patients with mild-to-moderate TBI that struggle with RTW due to persistent symptoms, i.e., those who present themselves to treatment clinics. Furthermore, the relatively modest sample size in this study (N = 87) limited the number of explanatory factors that could be assessed. Also, the measurement timeline (personality traits, PPCS, and RTW were all measured at 15 months post-injury) prevented us from performing additional tests of mechanisms of mediation, as mediation generally is considered a predictive model. Additionally, as we used the NEO-FFI-3, we were unable to investigate possible associations between the more fine-grained facets within each of the five personality dimensions and outcomes, which could have yielded more information on the influence of specific facet-level traits.

In our analysis, we focused on linear associations between personality traits, PPCS, and RTW. However, there might be non-linear associations worth investigating. For example, having low levels of a personality trait could be associated with similar outcomes as having high levels of a trait. In addition, to our knowledge, there are no studies examining combinations of different personality traits and recovery after mTBI. Interestingly, studies on other highly educated samples (medical students) have shown that a specific combination of personality traits (i.e., low extraversion, high neuroticism, and high conscientiousness) can predict stress [66]. It remains to be seen if these predictions also are valid in a mTBI sample, but combinations of different personality traits or ‘personality profiles’ should be the subject of further analysis regarding outcomes after mTBI, and we plan to look into this matter in future data analysis. This study did not find a significant association between relationship status and PPCS or RTW. Future studies may want to further investigate this relationship and that of other mechanisms related to the patient’s family and social context.

In conclusion, this study provides valuable information about the association between personality traits, symptom burden, and RTW after mild-to-moderate TBI. Assessing personality may be of value when patients with mild-to-moderate TBI are assessed for prolonged symptoms and when planning treatment, including psychological interventions and vocational rehabilitation, during the first year after injury. The results warrant greater awareness among clinicians and researchers regarding the impact of personality factors, including direct and indirect effects on PPCS, depressive symptoms, and RTW. PPCS and RTW after mild-to-moderate TBI seem to be related to and influenced by psychological mechanisms based on both symptoms (e.g., anxiety, depression, or emotional distress) and personality; thus, early identification of these psychological risk factors can help clinicians initiate more targeted interventions.

## Figures and Tables

**Table 1:** Baseline characteristics of the participants (N = 87).

		N *	% *
Sociodemographic factors	Age, mean (SD), y	43	(10)
	Sex, female	52	(60)
	Education, mean (SD), y	16	(2)
	Married/cohabitating	58	(67)
Cause of injury	Falls	38	(44)
	Exposure to inanimate objects	17	(19.5)
	Traffic accidents	15	(17)
	Sports	12	(14)
	Violence	4	(4.5)
	Unknown	1	(1)
Intracranial findings on CT/MRI	Yes	21	(24)
Injury severity	Mild	82	(94)
	Moderate	5	(6)
LOC	Yes	32	(37)
PTA	Yes	41	(38)
Extra-cranial injury	Yes	46	(53)
Work-related factors	Occupation, white-collar	80	(92)
	Employed in public sector	41	(47)
	Permanent position	80	(92)
	Full-time position	76	(87)
Sick leave at baseline	80–100%	73	(84)
	50–79%	14	(16)
Symptom burden	Total score on RPQ, mean (SD)	28	(11)
	Total score on PHQ-9, mean (SD)	9	(5)
	PHQ-9 score 10 or above	39	(44)
Personality traits, mean, (SD)	Neuroticism	49.2	(10.3)
	Extraversion	44.4	(10.7)
	Openness to experience	47.7	(9)
	Agreeableness	54	(9.4)
	Conscientiousness	55.7	(7.5)

Abbreviations: CT, computed tomography; LOC, loss of consciousness; MRI, magnetic resonance imaging; PTA, posttraumatic amnesia; RPQ, Rivermead Post-Concussion Symptoms Questionnaire; PHQ-9, Patient Health Questionnaire-9. * The values given are n (%) unless specified otherwise.

**Table 2:** Association between personality traits and PPCS * at 15 months post-injury (regression), N = 87.

Variable	Coeff.	S.E.	*p*-Value	C.I.
Female	8.17	2.16	**0.000**	3.93 to 12.40
Neuroticism	0.36	0.13	**0.005**	0.11 to 0.61
Openness to experience	0.31	0.10	**0.003**	0.11 to 0.51
Conscientiousness	0.62	0.14	**0.000**	0.34 to 0.90
Injury severity	−6.01	3.55	0.090	−12.95 to 0.94
Extra-cranial injury	4.59	2.10	**0.029**	0.47 to 8.72
Depressive symptoms	1.14	0.23	**0.000**	0.68 to 1.60
Intervention	1.76	2.00	0.379	−2.16 to 5.68
Constant	−60.19	11.89	0.000	−83.50 to −36.88
Wald chi2 (df.)		160.59 (8)		
Prob > Chi2		0.000		
R2		0.56		
Adj. R2		0.52		

* PPCS: Persistent post-concussive symptoms. Bold indicates statistical significance.

**Table 3:** Association between personality traits and RTW * at 15 months post-injury (regression), N = 87.

Variable	Step I				Step II			
	Coeff.	S.E.	*p*-Value	C.I.	Coeff.	S.E.	*p*-Value	C.I.
Female	−21.88	7.42	**0.003**	−36.42 to −7.35	−11.97	8.10	0.139	−27.85 to 3.91
Neuroticism	−1.19	0.45	**0.008**	−2.06 to −0.31	−0.75	0.50	0.134	−1.74 to 0.23
Openness to experience	−0.65	0.42	0.120	−1.47 to 0.17	−0.28	0.42	0.509	−1.10 to 0.55
Conscientiousness	−1.18	0.61	**0.050**	−2.37 to −0.002	−0.44	0.70	0.532	−1.81 to 0.93
Injury severity	−2.92	16.39	0.859	−35.04 to 29.21	−10.17	14.81	0.493	−39.20 to 18.87
Extra-cranial injury	0.92	8.12	0.910	−15.01 to 16.84	6.44	7.95	0.418	−9.14 to 22.03
Employed in public sector	14.95	7.20	**0.038**	0.82 to 29.07	14.67	6.94	**0.034**	1.07 to 28.26
Depressive symptoms	−0.43	0.91	0.634	−2.21 to 1.35	0.94	0.91	0.301	−0.84 to 2.72
Intervention	−13.99	7.36	0.057	−28.41 to 0.43	−11.85	7.36	0.107	−26.28 to 2.57
PPCS ** at 15 months post-injury	-	−	−	−	−1.21	0.46	**0.009**	−2.10 to −0.31
Constant	247.84	48.72	0.000	152.4 to 343.3	175.59	54.64	0.001	68.49 to 282.69
Wald chi2 (df.)	420.97 (9)				480.61 (10)			
Prob > Chi2	0.000				0.000			
R2	0.27				0.35			
Adj. R2	0.19				0.27			

* RTW: Return to work. ** PPCS: Persistent post-concussive symptoms. Bold indicates statistical significance.

## Data Availability

All datasets generated or analyzed during the current study are available from the corresponding author upon reasonable request.

## Abbreviations

CT	Computed tomography
FFM	The five-factor model of personality
GCS	Glasgow Coma Scale
LOC	Loss of consciousness
MRI	Magnetic resonance imaging
mTBI	Mild traumatic brain injury
NEO-FFI-3	The NEO Five-Factor Inventory-3
NEO-PI-3	The NEO Personality Inventory-3
OUH	Oslo University Hospital
PHQ-9	The Patient Health Questionnaire—9-item scale
PPCS	Persistent post-concussion symptoms
PTA	Posttraumatic amnesia
RCT	Randomized controlled trial
RPQ	Rivermead Post Concussion Symptoms Questionnaire
RTW	Return to work
TBI	Traumatic brain injury

## Appendix A

**Table A1:** Baseline characteristics of the participants included (N = 87) and excluded (N = 29) in this study.

		Included N = 87		Excluded N = 29		
		N *	% *	N *	% *	*p*-Value
Sociodemographic factors	Age, mean (SD), y	43	(10)	40	(9.5)	0.1
	Sex, female	52	(60)	17	(59)	0.913
	Education, mean (SD), y	16	(2)	15	(2.7)	0.002
	Married/cohabitating	58	(67)	19	(66)	0.91
Cause of injury	Falls	38	(44)	11	(38)	0.659
	Exposure to inanimate objects	17	(19.5)	6	(20.5)	
	Traffic accidents	15	(17)	8	(27.5)	
	Sports	12	(14)	2	(7)	
	Violence	4	(4.5)	2	(7)	
	Unknown	1	(1)	-	-	
Intracranial findings on CT/MRI	Yes	21	(24)	6	(21)	0.801
Injury severity	Mild	82	(94)	27	(93)	0.822
	Moderate	5	(6)	2	(7)	-
LOC	Yes	32	(37)	8	(29)	0.350
PTA	Yes	41	(38)	12	(41)	0.710
Extra-cranial injury	Yes	46	(53)	12	(41)	0.591
Work-related factors	Occupation, white-collar	80	(92)	23	(79)	0.062
	Employed in public sector	41	(47)	11	(38)	0.389
	Permanent position	80	(92)	25	(86)	0.360
	Full-time position	76	(87)	27	(93)	0.396
Sick leave at baseline	80–100%	73	(84)	21	(72)	0.513
	50–79%	14	(16)	8	(28)	-
Symptom burden	Total score on RPQ, mean (SD)	28	(11)	29	(10)	0.550
	Total score on PHQ-9, Mean (SD)	9	(5)	9.8	(5.1)	0.610
	PHQ-9 score 10 or above	38	(44)	12	(41)	0.746

Abbreviations: CT, computed tomography; LOC, loss of consciousness; MRI, magnetic resonance imaging; PTA, posttraumatic amnesia; RPQ, Rivermead Post-Concussion Symptoms Questionnaire; PHQ-9, Patient Health Questionnaire-9. * The values given are n (%) unless specified otherwise.

## References

[1] Cassidy J.D., Carroll L., Peloso P., Borg J., Von Holst H., Holm L., Kraus J., Coronado V. (2004). Incidence, risk factors and prevention of mild traumatic brain injury: Results of the who collaborating centre task force on mild traumatic brain injury. J. Rehabil. Med..

[2] Andelic N., Sigurdardottir S., Brunborg C., Roe C. (2008). Incidence of Hospital-Treated Traumatic Brain Injury in the Oslo Population. Neuroepidemiology.

[3] Voormolen D.C., Polinder S., von Steinbuechel N., Vos P.E., Cnossen M.C., Haagsma J.A. (2019). The association between post-concussion symptoms and health-related quality of life in patients with mild traumatic brain injury. Injury.

[4] Sigurdardottir S., Andelic N., Roe C., Jerstad T., Schanke A.-K. (2009). Post-concussion symptoms after traumatic brain injury at 3 and 12 months post-injury: A prospective study. Brain Inj..

[5] Polinder S., Cnossen M.C., Real R.G.L., Covic A., Gorbunova A., Voormolen D.C., Master C.L., Haagsma J.A., Diaz-Arrastia R., von Steinbuechel N. (2018). A Multidimensional Approach to Post-concussion Symptoms in Mild Traumatic Brain Injury. Front. Neurol..

[6] Iverson G.L., Lange R.T., Wäljas M., Liimatainen S., Dastidar P., Hartikainen K.M., Soimakallio S., Öhman J. (2012). Outcome from Complicated versus Uncomplicated Mild Traumatic Brain Injury. Rehabil. Res. Pract..

[7] Iverson G.L., Gardner A.J., Terry D.P., Ponsford J.L., Sills A.K., Broshek D.K., Solomon G.S. (2017). Predictors of clinical recovery from concussion: A systematic review. Br. J. Sports Med..

[8] Meares S., Shores E.A., Taylor A.J., Batchelor J., Bryant R.A., Baguley I.J., Chapman J., Gurka J., Dawson K., Capon L. (2008). Mild traumatic brain injury does not predict acute postconcussion syndrome. J. Neurol. Neurosurg. Psychiatry.

[9] Ponsford J., Cameron P., Fitzgerald M., Grant M., Mikocka-Walus A., Schönberger M. (2012). Predictors of postconcussive symptoms 3 months after mild traumatic brain injury. Neuropsychology.

[10] Meares S., Shores E.A., Taylor A.J., Batchelor J., Bryant R.A., Baguley I.J., Chapman J., Gurka J., Marosszeky J.E. (2011). The prospective course of postconcussion syndrome: The role of mild traumatic brain injury. Neuropsychology.

[11] Dischinger P.C., Ryb G.E., Kufera J.A., Auman K.M. (2009). Early Predictors of Postconcussive Syndrome in a Population of Trauma Patients with Mild Traumatic Brain Injury. J. Trauma Acute Care Surg..

[12] Lange R.T., Iverson G.L., Rose A. (2011). Depression Strongly Influences Postconcussion Symptom Reporting Following Mild Traumatic Brain Injury. J. Head Trauma Rehabil..

[13] Lange R.T., Brickell T., French L.M., Ivins B., Bhagwat A., Pancholi S., Iverson G.L. (2013). Risk Factors for Postconcussion Symptom Reporting after Traumatic Brain Injury in U.S. Military Service Members. J. Neurotrauma.

[14] Silverberg N.D., Gardner A.J., Brubacher J.R., Panenka W.J., Li J.J., Iverson G.L. (2015). Systematic Review of Multivariable Prognostic Models for Mild Traumatic Brain Injury. J. Neurotrauma.

[15] Cancelliere C., Kristman V.L., Cassidy J.D., Hincapié C.A., Côté P., Boyle E., Carroll L.J., Stålnacke B.-M., Nygren-de Boussard C., Borg J. (2014). Systematic Review of Return to Work After Mild Traumatic Brain Injury: Results of the International Collaboration on Mild Traumatic Brain Injury Prognosis. Arch. Phys. Med. Rehabil..

[16] Bloom B., Thomas S., Ahrensberg J.M., Weaver R., Fowler A., Bestwick J., Harris T., Pearse R. (2018). A systematic review and meta-analysis of return to work after mild Traumatic brain injury. Brain Inj..

[17] Forslund M.V., Arango-Lasprilla J.C., Roe C., Perrin P.B., Sigurdardottir S., Andelic N. (2014). Multi-level modelling of employment probability trajectories and employment stability at 1, 2 and 5 years after traumatic brain injury. Brain Inj..

[18] Gaudette É., Seabury S.A., Temkin N., Barber J., DiGiorgio A.M., Markowitz A.J., Manley G.T. (2022). TRACK-TBI Investigators Employment and Economic Outcomes of Participants with Mild Traumatic Brain Injury in the TRACK-TBI Study. JAMA Netw. Open.

[19] Vikane E., Hellstrøm T., Røe C., Bautz-Holter E., Aßmus J., Skouen J.S. (2016). Predictors for Return to Work in Subjects with Mild Traumatic Brain Injury. Behav. Neurol..

[20] Koning M.E. de, Scheenen M.E., Horn H.J. van der, Timmerman M.E., Hageman G., Roks G., Spikman J.M., Naalt J. (2017). van der Prediction of work resumption and sustainability up to 1 year after mild traumatic brain injury. Neurology.

[21] Evered L., Ruff R., Baldo J., Isomura A. (2003). Emotional Risk Factors and Postconcussional Disorder. Assessment.

[22] APA Dictionary of Psychology. https://dictionary.apa.org/.

[23] Costa P.T., McCrae R.R. (1992). Normal personality assessment in clinical practice: The NEO Personality Inventory. Psychol. Assess..

[24] Martinsen Ø.L., Nordvik H., Østbø L.E. (2011). The NEO PI-R in a North European Context. Scand. J. Organ. Psychol..

[25] McCrae R.R., Costa P.T., Del Pilar G.H., Rolland J.-P., Parker W.D. (1998). Cross-Cultural Assessment of the Five-Factor Model: The Revised NEO Personality Inventory. J. Cross-Cult. Psychol..

[26] Yamagata S., Suzuki A., Ando J., Ono Y., Kijima N., Yoshimura K., Ostendorf F., Angleitner A., Riemann R., Spinath F.M. (2006). Is the genetic structure of human personality universal? A cross-cultural twin study from North America, Europe, and Asia. J. Personal. Soc. Psychol..

[27] Israel S., Moffitt T.E., Belsky D.W., Hancox R.J., Poulton R., Roberts B., Thomson W.M., Caspi A. (2014). Translating personality psychology to help personalize preventive medicine for young adult patients. J. Personal. Soc. Psychol..

[28] (2006). Personality as Risk and Resilience in Physical Health-Timothy W. Smith. https://journals.sagepub.com/doi/abs/10.1111/j.1467-8721.2006.00441.x?journalCode=cdpa.

[29] Tackett J.L., Lahey B.B., Widiger T.A. (2017). Neuroticism. The Oxford Handbook of the Five Factor Model.

[30] Clarke L.A., Genat R.C., Anderson J.F.I. (2012). Long-term cognitive complaint and post-concussive symptoms following mild traumatic brain injury: The role of cognitive and affective factors. Brain Inj..

[31] Merz Z.C., Zane K., Emmert N.A., Lace J., Grant A. (2019). Examining the relationship between neuroticism and post-concussion syndrome in mild traumatic brain injury. Brain Inj..

[32] Skandsen T., Stenberg J., Follestad T., Karaliute M., Saksvik S.B., Einarsen C.E., Lillehaug H., Håberg A.K., Vik A., Olsen A. (2021). Personal Factors Associated with Postconcussion Symptoms 3 Months After Mild Traumatic Brain Injury. Arch. Phys. Med. Rehabil..

[33] Summerell P.A., Smillie L.D., Anderson J.F.I. (2021). Personality traits beyond Neuroticism predict post-concussive symptomatology in the post-acute period after mild traumatic brain injury in premorbidly healthy adults. Appl. Neuropsychol. Adult.

[34] Harvey A.G., Bryant R.A. (1998). Predictors of acute stress following mild traumatic brain injury. Brain Inj..

[35] Rush B.K., Malec J.F., Brown A.W., Moessner A.M. (2006). Personality and functional outcome following traumatic brain injury. Rehabil. Psychol..

[36] Parker H.A., Ranson J., McCrea M.A., Hoelzle J., deRoon-Cassini T., Nelson L.D. (2021). Personality Characteristics and Acute Symptom Response Predict Chronic Symptoms After Mild Traumatic Brain Injury. J. Int. Neuropsychol. Soc..

[37] (2020). Sickness Benefit (Sykepenger) for Employees. https://www.nav.no/en/home/benefits-and-services/Sickness-benefit-for-employees.

[38] Howe E.I., Langlo K.-P.S., Terjesen H.C.A., Røe C., Schanke A.-K., Søberg H.L., Sveen U., Aas E., Enehaug H., Alves D.E. (2017). Combined cognitive and vocational interventions after mild to moderate traumatic brain injury: Study protocol for a randomized controlled trial. Trials.

[39] Howe E.I., Fure S.C.R., Løvstad M., Enehaug H., Sagstad K., Hellstrøm T., Brunborg C., Røe C., Nordenmark T.H., Søberg H.L. (2020). Effectiveness of Combining Compensatory Cognitive Training and Vocational Intervention vs. Treatment as Usual on Return to Work Following Mild-to-Moderate Traumatic Brain Injury: Interim Analysis at 3 and 6 Month Follow-Up. Front. Neurol..

[40] Fure S.C.R., Howe E.I., Andelic N., Brunborg C., Sveen U., Røe C., Rike P.-O., Olsen A., Spjelkavik Ø., Ugelstad H. (2021). Cognitive and vocational rehabilitation after mild-to-moderate traumatic brain injury: A randomised controlled trial. Ann. Phys. Rehabil. Med..

[41] McCrae R.R., Costa P.T. (2007). Brief Versions of the NEO-PI-3. J. Individ. Differ..

[42] Martinsen Ø.L., Nordvik H., Østbø L.E. (2005). Norske versjoner av NEO PI-R og NEO FFI. Tidsskr Nor Psykologforening.

[43] Cifu D., Hurley R., Peterson M., Cornis-Pop M., Rikli P.A., Ruff R.L., Scott S.G., Sigford B.J., Silva K.A., Tortorice K. (2009). Clinical practice guideline: Management of Concussion/Mild Traumatic Brain Injury. J. Rehabil. Res. Dev..

[44] King N.S., Crawford S., Wenden F.J., Moss N.E.G., Wade D.T. (1995). The Rivermead Post Concussion Symptoms Questionnaire: A measure of symptoms commonly experienced after head injury and its reliability. J. Neurol..

[45] TBIDef_English_10-10.pdf. https://acrm.org/wp-content/uploads/pdf/TBIDef_English_10-10.pdf.

[46] Østbø L.E., Nordvik H. (2008). Personlighetsinventoriet NEO PI-R: Klinisk Validitet. Tidsskr Nor Psykologforening.

[47] Teasdale G., Jennett B. (1974). Assessment of coma and impaired consciousness: A Practical Scale. Lancet.

[48] Greenspan L., McLellan B.A., Greig H. (1985). Abbreviated Injury Scale and Injury Severity Score: A scoring chart. J. Trauma.

[49] Kroenke K., Spitzer R.L., Williams J.B.W. (2001). The PHQ-9. J. Gen. Intern. Med..

[50] StataCorp LLC (2019). Stata Statistical Software: Release 16.

[51] Yuen K.-M., Tsai Y.-H., Lin W.-C., Yang C.-C., Huang S.-J. (2016). Retrospectively evaluated preinjury personality traits influence postconcussion symptoms. Appl. Neuropsychol. Adult.

[52] Broshek D.K., De Marco A.P., Freeman J.R. (2015). A review of post-concussion syndrome and psychological factors associated with concussion. Brain Inj..

[53] Jackson J.J., Roberts B.W., Widiger T.A. (2017). Conscientiousness. The Oxford Handbook of the Five Factor Model.

[54] Wood A.M., Froh J.J., Geraghty A.W.A. (2010). Gratitude and well-being: A review and theoretical integration. Clin. Psychol. Rev..

[55] Boyce C.J., Wood A.M., Brown G.D.A. (2010). The dark side of conscientiousness: Conscientious people experience greater drops in life satisfaction following unemployment. J. Res. Personal..

[56] Kok A.A.L., Plaisier I., Smit J.H., Penninx B.W.J.H. (2017). The impact of conscientiousness, mastery, and work circumstances on subsequent absenteeism in employees with and without affective disorders. BMC Psychol..

[57] Sagstad K., Howe E.I., Fure S.C.R., Løvstad M., Enehaug H., Ugelstad H., Feiring M., Andelic N., Sveen U. (2023). Transition back to work after mild TBI: A qualitative study. Scand. J. Occup. Ther..

[58] Sutin A.R., Widiger T.A. (2017). Openness. The Oxford Handbook of the Five Factor Model.

[59] Cheng H., Furnham A. (2012). Childhood cognitive ability, education, and personality traits predict attainment in adult occupational prestige over 17years. J. Vocat. Behav..

[60] Mikolić A., van Klaveren D., Groeniger J.O., Wiegers E.J.A., Lingsma H.F., Zeldovich M., von Steinbüchel N., Maas A.I.R., Roeters van Lennep J.E., Polinder S. (2021). Differences between Men and Women in Treatment and Outcome after Traumatic Brain Injury. J. Neurotrauma.

[61] Fure S.C.R., Howe E.I., Andelic N., Brunborg C., Olsen A., Rike P.-O., Spjelkavik Ø., Enehaug H., Røe C., Løvstad M. (2023). Workplace Factors Associated with Return to Work After Mild-to-Moderate Traumatic Brain Injury. J. Head Trauma Rehabil..

[62] Fisker J., Hjorthøj C., Hellström L., Mundy S.S., Rosenberg N.G., Eplov L.F. (2022). Predictors of return to work for people on sick leave with common mental disorders: A systematic review and meta-analysis. Int. Arch. Occup. Environ. Health.

[63] Predictors of Six-Month Inability to Return to Work in Previously Employed Subjects after Mild Traumatic Brain Injury: A TRACK-TBI Pilot Study. https://journals.sagepub.com/doi/epub/10.1177/20597002211007271.

[64] Tate R.L. (2003). Impact of pre-injury factors on outcome after severe traumatic brain injury: Does post-traumatic personality change represent an exacerbation of premorbid traits?. Neuropsychol. Rehabil..

[65] Rush B.K., Malec J.F., Moessner A.M., Brown A.W. (2004). Preinjury Personality Traits and the Prediction of Early Neurobehavioral Symptoms Following Mild Traumatic Brain Injury. Rehabil. Psychol..

[66] Tyssen R., Dolatowski F.C., Røvik J.O., Thorkildsen R.F., Ekeberg Ø., Hem E., Gude T., Grønvold N.T., Vaglum P. (2007). Personality traits and types predict medical school stress: A six-year longitudinal and nationwide study. Med. Educ..

